# Mass Fatality Management following the South Asian Tsunami Disaster: Case Studies in Thailand, Indonesia, and Sri Lanka

**DOI:** 10.1371/journal.pmed.0030195

**Published:** 2006-06-06

**Authors:** Oliver W Morgan, Pongruk Sribanditmongkol, Clifford Perera, Yeddi Sulasmi, Dana Van Alphen, Egbert Sondorp

**Affiliations:** **1**Health Policy Unit, London School of Hygiene and Tropical Medicine, London, United Kingdom; **2**Department of Forensic Medicine, Faculty of Medicine, Chiang Mai University, Chaing Mai, Thailand; **3**Department of Forensic Medicine, University of Ruhuna, Galle, Sri Lanka; **4**World Health Organization, Banda Aceh, Indonesia; **5**Pan American Health Organization, Washington, District of Columbia, United States of America; Health Action International Asia PacificSri Lanka

## Abstract

**Background:**

Following natural disasters, mismanagement of the dead has consequences for the psychological well-being of survivors. However, no technical guidelines currently exist for managing mass fatalities following large natural disasters. Existing methods of mass fatality management are not directly transferable as they are designed for transport accidents and acts of terrorism. Furthermore, no information is currently available about post-disaster management of the dead following previous large natural disasters.

**Methods and Findings:**

After the tsunami disaster on 26 December 2004, we conducted three descriptive case studies to systematically document how the dead were managed in Thailand, Indonesia, and Sri Lanka. We considered the following parameters: body recovery and storage, identification, disposal of human remains, and health risks from dead bodies. We used participant observations as members of post-tsunami response teams, conducted semi-structured interviews with key informants, and collected information from published and unpublished documents.

Refrigeration for preserving human remains was not available soon enough after the disaster, necessitating the use of other methods such as dry ice or temporary burial. No country had sufficient forensic capacity to identify thousands of victims. Rapid decomposition made visual identification almost impossible after 24–48 h. In Thailand, most forensic identification was made using dental and fingerprint data. Few victims were identified from DNA. Lack of national or local mass fatality plans further limited the quality and timeliness of response, a problem which was exacerbated by the absence of practical field guidelines or an international agency providing technical support.

**Conclusions:**

Emergency response should not add to the distress of affected communities by inappropriately disposing of the victims. The rights of survivors to see their dead treated with dignity and respect requires practical guidelines and technical support. Mass fatality management following natural disasters needs to be informed by further field research and supported by a network of regional and international forensic institutes and agencies.

## Abstract 2

Case studies were conducted to systematically document how the bodies of those killed in the tsunami were managed in Thailand, Indonesia, and Sri Lanka. Many lessons can be learned, though more research is needed.

## Editors' Summary

**Background.:**

Some 226,408 people died in the tsunami that hit countries across South Asia on 26 December 2004. As well as providing assistance to the living, a crucially important part of the disaster relief effort was the recovery, identification, and disposal of the dead. However, there is very little consensus about the best way to handle and identify large numbers of bodies. Although natural disasters that kill many people occur frequently, most guidelines for the management of large numbers of dead bodies have come out of the experience gained from transport accidents and from terrorist incidents, and these guidelines are not directly relevant; for example, natural disasters often cause many more deaths than transport accidents or terrorist attacks. It is important for survivors that the bodies of the dead are handled with respect and that the dead are identified so that survivors know what has happened to missing relatives. However, at the same time many people are afraid of what the effect of many dead bodies might be on the living; one belief is that dead bodies are a source of disease. Such a belief can lead to the inappropriately rapid burial of bodies before identification has been done.

**Why Was This Study Done?:**

The tsunami of 2004 provided an opportunity to study four different aspects of how the dead were handled in a number of different countries: how the bodies were recovered, how the bodies were identified, how the bodies were disposed of, and what, if any, were the health effects of the large number of bodies on survivors. The authors wanted to then use the results to make recommendations for use in future natural disasters.

**What Did the Researchers Do and Find?:**

The authors interviewed in person, in writing, and by E-mail key people involved in the handling of the dead in three of the countries affected by the tsunami: Thailand (where 8,345 people died), Indonesia (where 165,708 people died), and Sri Lanka (where 35,399 people died). The authors discovered that there were a huge number of people and agencies involved in the handling of the dead; for example, in Indonesia 42 different organizations were involved in recovering bodies.

None of the countries had sufficient refrigerated storage available to store bodies until they could be identified. Some effective alternatives were used, such as temporary burial in shallow graves—where the temperature is lower than in the ambient air—with the intention of exhuming the bodies later for identification. However, many bodies were hurriedly buried in mass graves because they were decomposing; these bodies were almost impossible to identify.

Methods and efficiency of identification varied between and within countries. One hospital in Sri Lanka excelled by systematically photographing all bodies brought in and recording sex, height, and personal effects: 87% of the bodies brought here were identified. But in most areas rates of identification were much lower. It seemed that simple methods of identification were the most useful: photographs taken quickly before the bodies started to decompose, dental records, and personal effects found on the bodies. DNA analysis was only useful for a small number of bodies.

When it came to disposal of the bodies, again procedures differed widely, and in some cases were dictated by religious needs—for example, in some Muslim communities all bodies were buried within 24 hours, making counting and identification of the dead very difficult. Mass graves were often used, but these caused problems; for example, haphazard arrangement of the bodies meant that later exhumation and identification would be impossible.

The authors concluded that there was virtually no health impact of the dead bodies on survivors. Other studies found that there were no epidemics among the surviving population, and that most effects were on those who handled bodies in temporary morgues, where there were the expected variety of sharp-implement injuries and mucosal splashes with body fluids, along with heat stress and dehydration due to overuse of personal protective equipment such as respirators.

**What Do These Findings Mean?:**

How efficiently bodies were handled after the tsunami varied widely across and even within countries. The authors conclude that much of this variety was because of a lack of national or local plans for such mass fatalities, along with a lack of practical field guidelines. There was little coordination of all of the different organizations involved. However, in some places bodies were handled very well. The authors drew on their findings to suggest guidelines for the possible future management of large numbers of bodies, and also suggested that further research should be done. Reassuringly, the large numbers of bodies did not cause problems for the survivors, so in the future survivors should be encouraged to systematically identify the dead rather than rushing to bury them because of fear of disease.

**Additional Information.:**

Please access these Web sites via the online version of this summary at http://dx.doi.org/10.1371/journal.pmed.0030195.

• The World Heath Organization has a Web page that brings together much information on the tsunami and its aftermath

• News from the United Nations special envoy for the tsunami can be found on its Web site

• An article published by the Pan American Health Organization called “Disaster Myths That Just Won't Die”

• Field guidelines for managing mass fatality natural disasters developed by an international workshop following the tsunami

## Introduction

Globally, there are at least six natural disasters every year that kill more than 500 people [1]. Although management of human remains is one of the most difficult aspects of disaster response, there are currently no technical guidelines for dealing with large numbers of dead bodies following natural disasters. Existing methods developed for transport accidents and acts of terrorism are not directly transferable as they are designed for a smaller number of victims within a criminal or international medico-legal framework [2–4]. Developing appropriate guidelines for natural disasters is further complicated by the absence of information about post-disaster management of the dead following previous disasters.

Experience from the last 25 y suggests that a common reaction following mass fatality natural disasters is fear that dead bodies will cause epidemics [5,6]. This fear has frequently been used to justify rapid burial of human remains in mass graves with no identification [7]. Consequences of such mismanagement include increased psychological distress for survivors and legal problems affecting inheritance, compensation, insurance, and re-marriage of spouses [7–9]. Diplomatic tensions may also occur when foreign tourists are involved.

The tsunami disaster in South Asia on 26 December 2004 was one of the largest natural disasters in recent times (Table 1). Management of the dead varied remarkably between affected countries, with the biggest international forensic investigation in history following a natural disaster mounted in Thailand, while in other countries, local authorities were left to cope as best they could. The size of the disaster and the different responses provided an important opportunity to systematically document and learn about methods for managing human remains following large natural disasters. In this paper we present our findings from three case studies in Indonesia, Sri Lanka, and Thailand, and make recommendations for future disasters.

**Table 1:**
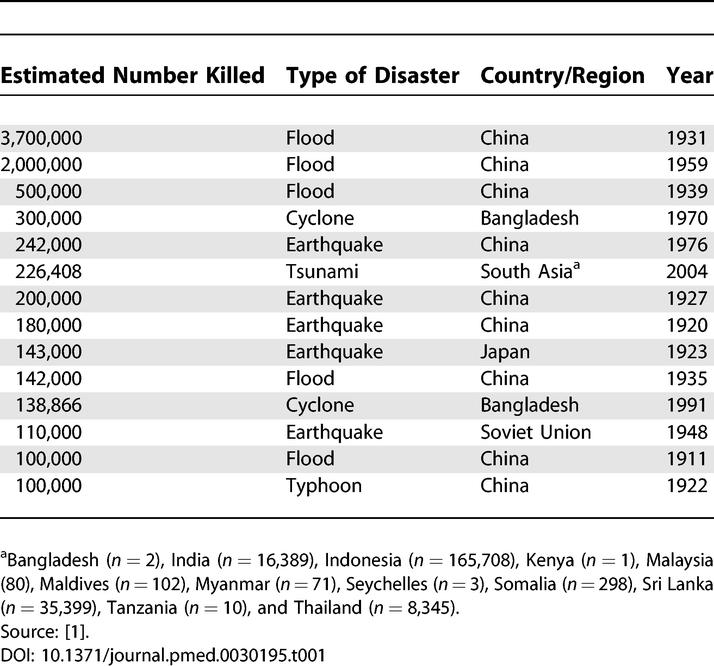
Natural Disasters That Have Caused at Least 100,000 Deaths between 1900 and 2005

## Methods

We used a descriptive multiple-case study design [10]. The study was deliberately designed to compare and contrast the management of a large number of fatalities in different countries affected by the tsunami. Each case was a different country. Our resources enabled us to select three countries. We therefore selected countries with (1) a large number of fatalities caused by the tsunami and (2) different levels of sophistication used to manage the dead.

At the beginning of the study we determined to examine four parameters: (1) methods of body recovery and storage, (2) methods of victim identification, (3) methods of disposal of human remains, and (4) public health issues associated with the management of a large number of dead bodies. Where possible, we used triangulation, whereby data were sought from different sources to supplement and validate observations. Several authors (P. S., C. P., Y. S., and D. V. A.) made participant observations while working as members of post-tsunami response teams in the affected countries. Semi-structured interviews using a checklist/question prompt were conducted with key informants by one of the authors (O. W. M.) between 18 February and 4 March 2005. Purposive sampling [11] was used to select individuals with operational and managerial responsibility for the management of the dead. Where face-to-face interviews were not possible, interviews were conducted by telephone or E-mail. Interviews were conducted in English or with the aid of an interpreter recruited in each country specifically for the study. In each country we sought published and unpublished documents (situation reports, official statistics, evaluation reports, technical documents, guidelines for victim identification, and public health reports) from national ministries of health and government offices, the World Health Organization (WHO), non-governmental organisations, and voluntary groups. We analysed field and interview notes thematically and inductively (generating ideas from the data), using the study parameters as a framework for analysis [10,11].

## Results

We selected Thailand, Sri Lanka, and Indonesia for this study. The number of fatalities in each of these countries has been estimated as 8,345, 35,399, and 165,708, respectively [1]. Participant observers (P. S., C. P., Y. S., and D. V. A.) spent at least 4 wk working in affected areas. Interviews were conducted with 40 key informants from the voluntary sector (*n =* 9), ministries of health (*n =* 8), military (*n =* 6), WHO (*n =* 5), police (*n =* 5), hospital staff (*n =* 4), and government officials (*n =* 3). Reviewed documents included WHO situation reports (*n =* 37) [12], evaluation or surveillance reports (*n =* 4), and technical documents (*n =* 4).

### Body Recovery and Storage

**Figure 1:**
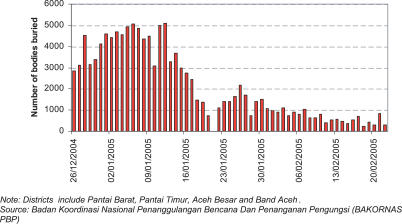
Daily Number of Bodies Buried in Banda Aceh and Surrounding Areas, 26 December 2004 to 22 February 2005 Districts include Pantai Barat, Pantai Timur, Aceh Besar, and Band Aceh. (Source: Badan Koordinasi Nasional Penanggulangan Bencana Dan Penanganan Pengungsi—BAKORNAS PBP)

Body recovery is the first phase of the management of dead bodies. In all countries it was characterised as being initially chaotic and uncoordinated, involving a large number of different actors. In Thailand, body recovery was done by foreign tourists, local volunteers, Thai non-governmental organisations that specialise in body recovery following disasters (Po-Tek-Tung Foundation and Ruam-Ka-Tan-Yu Foundation), the military, and the police. In Indonesia, the body recovery phase lasted several months (Figure 1), and, under the coordination of the military, 42 different organisations were involved. In Sri Lanka, body recovery was done almost exclusively by the affected communities themselves. In all cases, bodies were taken to multiple locations, and relatives did not know where their family members had been taken.

None of the countries had sufficient refrigerated storage immediately available. In Thailand, the only country able to mobilise large numbers of refrigerated containers, it took about 2 wk to provide about 100 containers needed to store around 3,600 bodies. Temporary burial in shallow trench graves (about 1 m deep) was used effectively to store about 600 bodies. Effective use of dry ice proved difficult: when placed on top of the bodies it damaged them because of its low temperature, while not providing sufficient overall cooling to stop decomposition. Handling large quantities of dry ice also caused many skin burns among individuals handling it [13]. However, it was found that an effective method was to build a small wall of dry ice surrounding a group of bodies, and then to cover the group with a tent or tarpaulin.

### Identification

Victim identification differed considerably between the three countries. In Indonesia, simple visual identification was attempted in the first few days. However the sheer number of bodies meant that it was impossible to arrange viewing for all bodies or store the bodies for later identification. Nevertheless, the body recovery teams successfully identified over 500 victims using personal effects such as identity cards and jewellery, and even mobile telephone SIM cards.

In Sri Lanka, the Centre for National Operations (an ad hoc governmental disaster management committee) mandated that local authorities take photographs and collect fingerprints of all the victims. However, because of damaged communications infrastructure, these instructions only arrived after 2 or 3 d, by which time decomposition had distorted facial features. In all, many hundreds of photographs were taken by police photographers, medical staff, journalists, and freelance photographers. In some cases, the films were not developed as there were insufficient funds to pay either the developer or freelance photographers. There were, however, outstanding examples, such as the hospital at Matara, where digital photographs were taken and basic information recorded (sex, height, and personal effects) for each body as it was brought into the hospital. Over 87% of the 547 victims handled by the hospital were identified [14]. Foreign victims, largely found in the eastern part of the country, were sent directly to the capital city Colombo, where an Identification Centre was established with support from the British government. During 2005, the Identification Centre also supervised six major exhumations to search for missing foreigners who were buried along with Sri Lankan nationals. A total of 155 bodies were examined by the disaster victim identification team. Analysis of DNA and dental records was used to successfully identify these individuals, who came from 18 different countries.

On 27 December, the first Thai forensic teams, many travelling independently under their institutes, started arriving in the affected areas of southern Thailand. They rapidly set up basic identification facilities in local temples. During the first 7–10 d of operations, Thai forensic teams examined around 3,600 bodies. The examination included external examination, photography, and recording of all personal effects. Fingerprints were taken from about 600 cadavers. DNA samples were collected from almost all bodies during the first few days, and included hair and soft tissues and, later, ribs and teeth. During this initial phase, Thai forensic teams identified about 1,100 human remains and released them to the families. In addition, about 500 bodies were identified and released to relatives by local physicians and police without the support of forensic specialists.

After the first week, forensic teams from other countries started to arrive in Thailand. They formed an international disaster victim identification committee to work in collaboration with the Royal Thai Police [15]. In Phuket, the committee's information centre was established with the financial support of the Australian government. The Thai government decided to combine the efforts of the Thai forensic experts, the Thai Royal Police, and international disaster victim identification committee teams, and on 13 January the Thai Tsunami Victim Identification (TTVI) centre was established in Phuket [16]. In collaboration with Interpol, the TTVI established a central mortuary in Phuket, sponsored by the Norwegian government. It was decided to examine or re-examine all 3,777 remaining victims using Interpol's standard protocol [17]. This included external examination, personal effects, photographs, fingerprints, forensic pathological examination, dental examination, and DNA sampling from bone and teeth. As of 27 July 2005, 7 mo after the disaster, TTVI had identified 2,010 victims, with over 1,800 cadavers remaining unidentified [18]. Sixty-one percent of victims were identified by TTVI using dental examinations (*n =* 1,235), 19% using fingerprint records (*n =* 378), 1.3% using DNA analysis (*n =* 26), and 0.3% using physical evidence (*n =* 6). In a further 18% of cases (*n =* 365), more than one type of evidence was used [19].

### Disposal of Human Remains

In Thailand, unidentified victims were stored in refrigerated containers during identification activities. Bodies that were identified were disposed of by cremation or burial according to local custom. Bodies of foreign victims were repatriated by their respective embassies. Around Banda Aceh, Indonesia, there were 14 mass graves, the largest, at Lambarro, reportedly containing 60,000–70,000 victims. Finding suitable government land for these large graves was difficult, and in some instances graves were sited very close to communities. In the areas surrounding Banda Aceh, smaller village-level graves were often used. Many were constructed rapidly, sometimes within the village itself. This has caused difficulty for returning survivors wanting to exhume and re-locate the graves to outside the village. In many rural areas, there was no formal body recovery and disposal of remains. In Sri Lanka, most human remains were buried after 3 or 4 d. Common graves, in which bodies were buried haphazardly in several layers, were sited largely within existing cemeteries. However, within some Muslim communities the deceased were buried within the first 24 h according to custom, making it difficult for the local authorities to identify and count the dead. Additionally, there were concerns that some of the deceased, who were buried as Muslims, may have been from other religious groups.

### Health Impact from Dead Bodies

Shortly after the tsunami, WHO and national governments established early warning disease surveillance. No epidemics among the surviving populations were identified in the weeks after the tsunami [20]. In Banda Aceh, Indonesia, it took some 2 mo to bury the thousands of bodies (Figure 1). In spite of the prolonged presence of dead bodies, no epidemics occurred [21]. Among individuals handling human remains (recovery, identification, and disposal), we did not identify any reports of “occupational” infections. A health and safety assessment of temporary morgues in Thailand by the United States Centers for Disease Control and Prevention and the Thai Ministry of Public Health reported sharp-implement injuries and mucosal splashes with body fluids as well as heat stress and dehydration due to overuse of personal protective equipment such as respirators [13]. A questionnaire survey conducted by the Thai Ministry of Public Health of around 200 individuals involved in body recovery did not identify any reports of infectious disease (S. Sirituttanapruk, personal communication). Back injuries, caused by lifting bodies into trucks, were reported by Indonesian military. Body recovery teams faced potential injury risk from working among debris, especially from earthquake-damaged buildings. In Sri Lanka, most dead bodies were taken to local hospitals, which had an indirect health impact by disrupting the provision of medical assistance to survivors and threatening to close hospitals because of the smell of decomposition.

### Coordination and Preparedness

In each country, a large number of individuals and organisations were involved in managing the dead. Body recovery involved the affected community, voluntary organisations, the police, and the military. Doctors, medical staff, and forensic specialists were involved in death certification and collecting post-mortem data. National police forces and consulates or embassies were involved in collecting ante-mortem data (information about the deceased collected before death, such as dental or fingerprint records). Disposal of the bodies was done by the military or police, who also had legal responsibility for victim identification. No single person or organisation had a clear mandate to coordinate the process of collecting, identifying, and disposing of the dead, either nationally or locally. None of the countries had mass fatality plans.

## Discussion

The technical and logistical challenges of recovering and identifying victims after the tsunami were exceptional. The hot climate increased the rate of decomposition: bloating and discolouration of the human face rendered visual identification almost impossible after 24–48 h. Odours from decomposition caused concern about epidemics, and led local communities and national authorities to sanction mass (unplanned) burial without identification. Refrigeration for preserving human remains was not available soon enough, and no country had sufficient forensic capacity to identify thousands of victims. Lack of national or local mass fatality plans further limited the quality and timeliness of response, as did the absence of practical field guidelines or an international agency providing technical support.

### Strengths and Limitations

Unlike study designs that make statistical inferences about a population, case study designs are suitable for describing and understanding why events occur and for generating hypotheses for future study. Therefore, rather than select cases to be “representative”, we selected cases to highlight a range of experience. A case study design was especially appropriate in this situation because we had no previous information about how the management of mass fatalities is undertaken following natural disasters (and hence no a priori hypotheses to test).

Conducting research during a humanitarian emergency presents many challenges. For example, individuals from relief agencies and governmental bodies have heavy workloads and are under considerable stress. Consequently, allocating time to participate in research activities may be of secondary importance. The stressful nature of disaster response leads to a high turnover of staff, and some of the key informants were no longer available for interview during our fieldwork. We attempted to contact these individuals by telephone and E-mail, but this was not always possible. Finally, we found that the management of dead bodies was politically very sensitive, both at local and national government levels. For these reasons, it is likely that some key informants were not included.

### Storage, Identification, and Burial

Cold storage is vital for preserving evidence for identification. None of the countries could quickly mobilise sufficient refrigerated containers after the tsunami, and in Thailand, where refrigerated containers did eventually become available, most of the bodies had decomposed considerably by that time. The use of dry ice was reasonably effective, but it was difficult to manage, logistically intensive, and a significant cause of work-related injury. An alternative is normal ice (frozen water), as used after the Bali bombing in 2002 [22]. However, large quantities of melted water are produced that contain products of decomposition, which are likely to create additional management problems [22,23]. For large numbers of dead bodies, the most practical option is temporary burial in trench graves. The temperature underground is lower than at the surface, and burial acts as “natural refrigeration”. At 1.2 m depth, bodies have been well preserved for several months [24]. However, this approach must include careful recording of the location of each body and good communications with the public and media, who may mistakenly interpret this as disposal of victims without identification.

The simplest form of identification used after the tsunami was visual recognition and photographs of fresh bodies. In the absence of cold storage, this needs to be done rapidly. After 24–48 h without cooling, gases build up within the body, swelling the face and lips and forcing the tongue out of the mouth, making visual identification unreliable. The epidermis detaches from the body, leaving un-pigmented skin, giving the appearance of a white cadaver, even in dark-skinned individuals [25]. Further, while visual identification is relatively simple, it will result in some misidentification. Injuries to the body, or the presence of blood, fluids, or dirt, especially around the head, will reduce the chance of correct recognition. Following the Bali bombing, visual identification was incorrect in about one-third of victims [22]. The effectiveness of this method following natural disasters is unknown, although reports from one hospital in Sri Lanka suggest that it can have good results [14].

Forensic techniques such as dental, fingerprint, and DNA analysis are effective because they can identify decomposed or damaged bodies. However, for large disasters they require many trained specialists and are resource intensive. Most importantly, these methods are only useful if comparative data are available. While fingerprint data are recorded for Thai citizens when identity cards are issued, and many Western victims had dental records, comparative data may be scarce in many parts of the world. Few countries have the capacity for DNA collection and analysis following large natural disasters. DNA identification is expensive, technically demanding, and logistically difficult to implement on a large scale [2]. In the case of the tsunami in Thailand, it proved to be a relatively unimportant method of identification. DNA identification should not be considered as a first-line method of identification, but rather should only be implemented when physical, fingerprint, and dental methods have been unsuccessful [26].

Communal burial may be necessary when the number of human remains is large, as happened in Sri Lanka and Indonesia. Haphazard commingling of cadavers in mass graves makes future exhumations extremely difficult. Communal graves should be clearly marked, with bodies well organised and buried in one layer. All affected countries had difficulty finding locations for graves while considering the wishes of the local community, access for relatives, and land ownership. Although few cremations took place in the countries studied, they should be avoided because they make identification exceptionally difficult, require large amounts of fuel, and rarely achieve complete incineration, necessitating burial of partially burned cadavers.

### Health Risks

The fear that dead bodies will cause epidemics among survivors, often encouraged by the media, prejudices proper handling and identification [6,27]. The unpredictable and chaotic nature of disasters means epidemiological evidence about associated infections is unavailable. A risk assessment suggests that the risk is small for members of the public and is primarily due to diarrhoea from drinking water contaminated with faecal matter from dead bodies [28]. This assessment of low risk, along with anecdotal observations over the last 20 y [6] and the absence of outbreaks in Banda Aceh despite the presence of several thousand bodies, should be considered the most convincing evidence to date that dead bodies pose a negligible threat to the general public after natural disasters.

Individuals who handle the dead (recovery, identification, and disposal) may be exposed to blood, body fluids, or faeces that contain chronic infections such as hepatitis B and C, HIV, tuberculosis, and gastrointestinal pathogens [28]. Simple precautions such as wearing gloves and washing hands will reduce transmission and hence reduce risks considerably. We did not identify any reports of “occupational” infections among body handlers. However, considering the relatively long incubation period for blood-borne infections and the low likelihood of testing among these individuals, it may have been too early to detect their incidence. Long term follow-up of this group is needed.

### Coordination and Preparedness

None of the countries had a single organisation with jurisdiction for recovery, identification, and disposal of bodies. Not only did this cause tension, but also added to the confusion and stress of relatives searching for family members. The lack of mass fatality plans meant that these issues had to be worked out during the response.

### Recommendations and Conclusions

The South Asian tsunami in 2004 was an extreme natural event resulting in many thousands of fatalities. Several important lessons can be highlighted for future disasters (Box 1). Until now, the failure to document and learn following mass fatality natural disasters means that similar mistakes occur time and time again. In May 2005, WHO, the Pan American Health Organization, and the International Committee of the Red Cross/Red Crescent convened an international workshop in the city of Lima, Peru, to share the experience of the tsunami and other previous disasters and to develop a first responders' manual for mass fatality natural disasters. These practical field guidelines were published in April 2006 [29].

Management of the dead has important socio-cultural implications, and emergency response should not add to the distress of affected communities through inappropriate handling and disposal of the victims. Promoting the rights of the survivors to see their dead treated with dignity and respect requires guidelines and technical support, which must be informed by further field research (Box 2). Moreover it is important that the international community promotes the rights of victims and communities by including standards for the management of the dead in existing humanitarian Sphere Project guidelines [30] (the Sphere Project is a collaboration of over 400 organisations that agree on minimum standards in disaster relief). Finally, no country has sufficient capacity to respond to very large disasters, and networks of countries, forensic institutes, and international agencies such as Interpol and WHO are needed to provide assistance for the management of the dead following future disasters.

**Box 1. Recommendations for the Management of the Dead after Natural Disasters**

Health Impacts

 • The health risk to the general public of large numbers of dead bodies is negligible

 • Drinking water must be treated to avoid possible diarrhoeal diseases

 • Body handlers should follow universal precautions for blood and body fluids, wear gloves, and wash their hands

Body Storage

 • Refrigerated containers provide the best storage, if available

 • Temporary burial in trench graves can be used if refrigeration is not available

Body Identification

 • Visual recognition or photographs of fresh bodies are the simplest forms of non-forensic identification and should be attempted after all natural disasters

 • If resources and comparative data are available, simpler methods can be supplemented by forensic techniques (dental, fingerprint, and DNA analysis)

Body Disposal

 • Communal graves may be necessary following large disasters

 • Bodies should be buried in one layer to facilitate future exhumation

 • Graves should be clearly marked

Coordination

 • A named person/organisation should have an agreed mandate to coordinate the management of dead bodies

Preparedness

 • Mass fatality plans should be included in national and local disaster preparedness activities

 • Systematic documentation about how the dead are managed in future disasters is needed to learn from them

Communications

 • Close working with the media is needed to avoid misinformation and to promote the rights of the survivors to see their dead treated with dignity and respect

**Box 2. Areas of Further Research in the Management of Dead Bodies following Natural Disasters**

 • Different methods of body storage where refrigeration is not available.

 • Hydrological characteristics of mass communal burial and measures to avoid groundwater contamination.

 • Epidemiological studies of infectious and non-infectious health risks for individuals recovering and identifying dead bodies.

 • Methods for victim identification in situations where specialist forensic support is limited or unavailable, especially using visual and fingerprint identification.

 • Strategies for developing regional and international forensic capacity and resources.

 • Systems and protocols for managing information about the dead and missing.

 • Social and cultural impacts of bereavement and the imperative to identify missing relatives and friends.

 • Social and cultural acceptability of technical approaches for identification.

 • Community-level approaches to disaster preparedness and response with regard to the management of the dead.

## Abbreviations

TTVI: Thai Tsunami Victim Identification
WHO: World Health Organization
